# Using G6PD tests to enable the safe treatment of *Plasmodium vivax* infections with primaquine on the Thailand-Myanmar border: A cost-effectiveness analysis

**DOI:** 10.1371/journal.pntd.0005602

**Published:** 2017-05-24

**Authors:** Angela Devine, Minnie Parmiter, Cindy S. Chu, Germana Bancone, François Nosten, Ric N. Price, Yoel Lubell, Shunmay Yeung

**Affiliations:** 1 Mahidol-Oxford Tropical Medicine Research Unit, Mahidol University, Bangkok, Thailand; 2 Centre for Tropical Medicine and Global Health, Nuffield Department of Clinical Medicine, University of Oxford, Oxford, United Kingdom; 3 Boyd Orr Centre, University of Glasgow, Glasgow, United Kingdom; 4 Shoklo Malaria Research Unit, Mahidol-Oxford Tropical Medicine Research Unit, Mahidol University, Mae Sot, Thailand; 5 Global and Tropical Health Division, Menzies School of Health Research and Charles Darwin University, Darwin, Australia; 6 Faculty of Infectious and Tropical Disease, The London School of Hygiene & Tropical Medicine, London, United Kingdom; Tulane University School of Public Health and Tropical Medicine, UNITED STATES

## Abstract

**Background:**

Primaquine is the only licensed antimalarial for the radical cure of *Plasmodium vivax* infections. Many countries, however, do not administer primaquine due to fear of hemolysis in those with glucose-6-phosphate dehydrogenase (G6PD) deficiency. In other settings, primaquine is given without G6PD testing, putting patients at risk of hemolysis. New rapid diagnostic tests (RDTs) offer the opportunity to screen for G6PD deficiency prior to treatment with primaquine. Here we assessed the cost-effectiveness of using G6PD RDTs on the Thailand-Myanmar border and provide the model as an online tool for use in other settings.

**Methods/Principal findings:**

Decision tree models for the management of *P*. *vivax* malaria evaluated the costs and disability-adjusted life-years (DALYs) associated with recurrences and primaquine-induced hemolysis from a health care provider perspective. Screening with G6PD RDTs before primaquine use was compared to (1) giving chloroquine alone and (2) giving primaquine without screening. Data were taken from a recent study on the impact of primaquine on *P*. *vivax* recurrences and a literature review. Compared to the use of chloroquine alone, the screening strategy had similar costs while averting 0.026 and 0.024 DALYs per primary infection in males and females respectively. Compared to primaquine administered without screening, the screening strategy provided modest cost savings while averting 0.011 and 0.004 DALYs in males and females respectively. The probabilistic sensitivity analyses resulted in a greater than 75% certainty that the screening strategy was cost-effective at a willingness to pay threshold of US$500, which is well below the common benchmark of per capita gross domestic product for Myanmar.

**Conclusions/Significance:**

In this setting G6PD RDTs could avert DALYs by reducing recurrences and reducing hemolytic risk in G6PD deficient patients at low costs or cost savings. The model results are limited by the paucity of data available in the literature for some parameter values, including the mortality rates for both primaquine-induced hemolysis and *P*. *vivax*. The online model provides an opportunity to use different parameter estimates to examine the validity of these findings in other settings.

## Author summary

A single infection with *Plasmodium vivax* can cause multiple episodes of illness due to dormant liver parasites called hypnozoites. Primaquine is the only drug currently available to treat hypnozoites but is under-used because it can cause life-threatening red blood cell damage in people who have an inherited condition called glucose-6-phosphate dehydrogenase (G6PD) deficiency. In other locations, primaquine is given without testing for G6PD deficiency, putting patients at risk of potentially fatal hemolysis. New rapid diagnostic tests provide the opportunity to screen for G6PD deficiency prior to giving patients primaquine. Our study describes a cost-effectiveness analysis conducted using data gathered from the Thailand-Myanmar border. Our results show that screening for G6PD deficiency followed by primaquine treatment provided a few days of disability-free health per patient treated. This was achieved for similar costs as not giving primaquine to anyone or cost savings when compared to giving primaquine without screening. In addition to the health gains provided to patients, the safe use of primaquine will be a critical tool to eliminate malaria. We provide an interactive cost-effectiveness tool online that can be adapted to other locations to examine the potential costs and benefits of using rapid diagnostic tests for G6PD in different scenarios.

## Introduction

*Plasmodium vivax* is an important public health concern, particularly in Asia and South America, where it is now responsible for the majority of malaria cases. While traditionally regarded as a benign disease, *P*. *vivax* malaria has been associated with severe and fatal outcomes [1, 2]. As countries move toward malaria elimination and the overall incidence of malaria declines, the proportion of cases that are due to *P*. *vivax* infections increases [3, 4]. A single infection of *P*. *vivax* can lead to multiple relapses due to its ability to form dormant liver stage parasites called hypnozoites. These relapses are indistinguishable from new infections and repeated episodes can lead to a cumulative risk of anemia and malnutrition [5, 6]. In short latency relapse settings, the majority of *P*. *vivax* cases are thought to be due to relapses [7].

Primaquine is the only drug currently licensed for the radical cure of *P*. *vivax*; however, it can cause severe hemolysis in individuals with glucose-6-phosphate dehydrogenase (G6PD) deficiency, a common genetic disorder [8] that is positively associated with *P*. *vivax* incidence [9]. The prevalence of G6PD deficiency varies from less than 1% to more than 30%, with a mean of 8% in countries where malaria is endemic; equivalent to 350 million people worldwide [10]. G6PD deficiency is largely asymptomatic until individuals are exposed to oxidative stress from an external source, including certain drugs, such as primaquine, and foods, most notably fava beans [9]. The degree of enzyme deficiency varies widely depending upon the genotypic variant which varies with geographical region. A recent review found only 14 documented deaths attributable to primaquine use [11]; however, fatalities may have gone unreported [12].

The WHO recommends that primaquine be used for the radical cure of *P*. *vivax* infected patients who can be tested for G6PD deficiency [13]. The gold standard for diagnosing G6PD deficiency is the spectrophotometric assay, a test that requires a laboratory setting and specialized staff [14, 15]. The Fluorescent Spot Test (FST), which is the most widely used assay for G6PD deficiency, is easier to perform but requires basic laboratory equipment, electricity and a cold chain, rendering it difficult to use in remote settings. Thus, routine testing for G6PD deficiency prior to prescribing primaquine generally is not part of antimalarial policy in most countries [16]. Recently, the CareStart G6PD (Access Bio, Somerset, NJ, USA) lateral flow rapid diagnostic test (RDT) has become available for point of care testing. This phenotypic test has high sensitivity for an enzyme activity cut off of 30% [17, 18]; hence false negative results would rarely lead to a G6PD deficient individual receiving primaquine with an attendant risk of hemolysis. Unlike the other G6PD RDT by BinaxNOW (Alere, Orlando, FL, USA), the CareStart RDT can be used in settings where the temperature is above 25°C, a common necessity in *P*. *vivax* endemic settings[19, 20].

The availability of point of care G6PD tests is of clinical and public health importance so that *P*. *vivax* patients have safe access to primaquine treatment for the prevention of relapses and the resulting health complications [20]. Here we evaluate the cost-effectiveness of using G6PD RDTs on the Thailand-Myanmar border and present our model as an interactive web tool that can be adapted to other settings.

## Methods

### Base case analysis

A cost-effectiveness analysis [21] using a health care provider perspective was conducted with decision tree models for *P*. *vivax* infections using R statistical software [22] over a 1 year time horizon. The model was parameterized for the north-western border of Thailand with Myanmar (Tak Province), with the benefit of data on recurrences from a recent clinical trial at the Shoklo Malaria Research Unit (SMRU), which provides free of charge care to migrants and refugees [23] (S1 Appendix). In this population of migrants and refugees, the prevalence of G6PD deficiency was documented to be 9–18% [24]. The most common genetic variant was the Mahidol variant (88%) with Chinese-4, Viangchan, Açores, Seattle, and Mediterranean variants also present [24]. Low, unstable *P*. *vivax* transmission is seen in this area [3] with a frequent relapse pattern [25]. In recent years, the overall number of malaria cases has been decreasing while the prevalence of *P*. *vivax* in the population has remained relatively stable at 9% [3]. Routine practice along the border is to administer 14 days of supervised therapy with or without G6PD screening to patients able to attend the clinic; in practice this is a small proportion of the patients.

The testing of G6PD status with CareStart G6PD RDT before administering primaquine (“screening strategy”) was compared to a strategy in which no G6PD test is performed and primaquine is not used at all (“chloroquine strategy”). In addition, the screening strategy is compared with a strategy where primaquine is given to all patients without testing for G6PD deficiency (“primaquine strategy”) (Table 1). The chloroquine strategy and primaquine strategy were not directly compared to each other because (1) it is unlikely that in settings where the chloroquine strategy is used switching to the primaquine strategy would be a viable option due to the evident concerns about safety and (2) it is unlikely that settings where the primaquine strategy is used would consider changing to the chloroquine strategy due to its inability to achieve radical cure.

**Table 1:** Summary of key components of each strategy.

	Chloroquine strategy	Primaquine strategy	Screening strategy
**INITIAL VISIT**			
Treatment	Chloroquine given to all.	Chloroquine and supervised primaquine therapy (14 day) given to all. All individuals with severe G6PD deficiency stop primaquine therapy early. Females with intermediate G6PD deficiency who do not have severe hemolysis complete primaquine therapy.	Chloroquine and supervised primaquine therapy (14 day) given to those who test G6PD normal. Chloroquine and weekly primaquine given to those who test G6PD abnormal. Individuals with severe G6PD deficiency who are inadvertently given 14 day primaquine will stop therapy early. Females with intermediate G6PD deficiency who do not have severe hemolysis complete primaquine therapy.
**RECURRENCES (all costs and DALYs are weighted by the proportion of severe cases)**			
Costs	Cost per recurrence.	Cost per recurrence. Supervised primaquine (14 day) for those who are G6PD normal.	Cost per recurrence. Includes G6PD RDT. Supervised primaquine (14 day) for those who are G6PD true normal. Supervised primaquine (8 weekly) for those who test G6PD abnormal and false normal.
Effects	All have the number of recurrences for chloroquine treatment.	G6PD abnormal individuals have the number of recurrences for chloroquine treatment. G6PD normal individuals have the number of recurrences for primaquine treatment.	Those who test G6PD false normal have the number of recurrences for chloroquine treatment. All other individuals have the number of recurrences for primaquine treatment.

Recurrences were recorded over a one year time period in patients who were treated with chloroquine alone, as compared with those who were treated with chloroquine plus 14 days of supervised primaquine for each *P*. *vivax* episode (0.5 milligrams (mg)/kilogram (kg)/day) [23]. The relative risk of having at least one recurrence following primaquine treatment was 0.22 as compared to those receiving chloroquine alone. For those who had at least one recurrence, the mean number of recurrences was 3.54 in the chloroquine arm and 1.16 in the primaquine arm. The model applied the inclusion criteria of the clinical trial, which was restricted to patients who were six months and older, not pregnant and presenting with uncomplicated *P*. *vivax* malaria [23]. The mean age in the clinical trial was 21 years; this was used for the disability-adjusted life-year (DALY) calculations for years of life lost (S1 Appendix).

**Fig 1:**
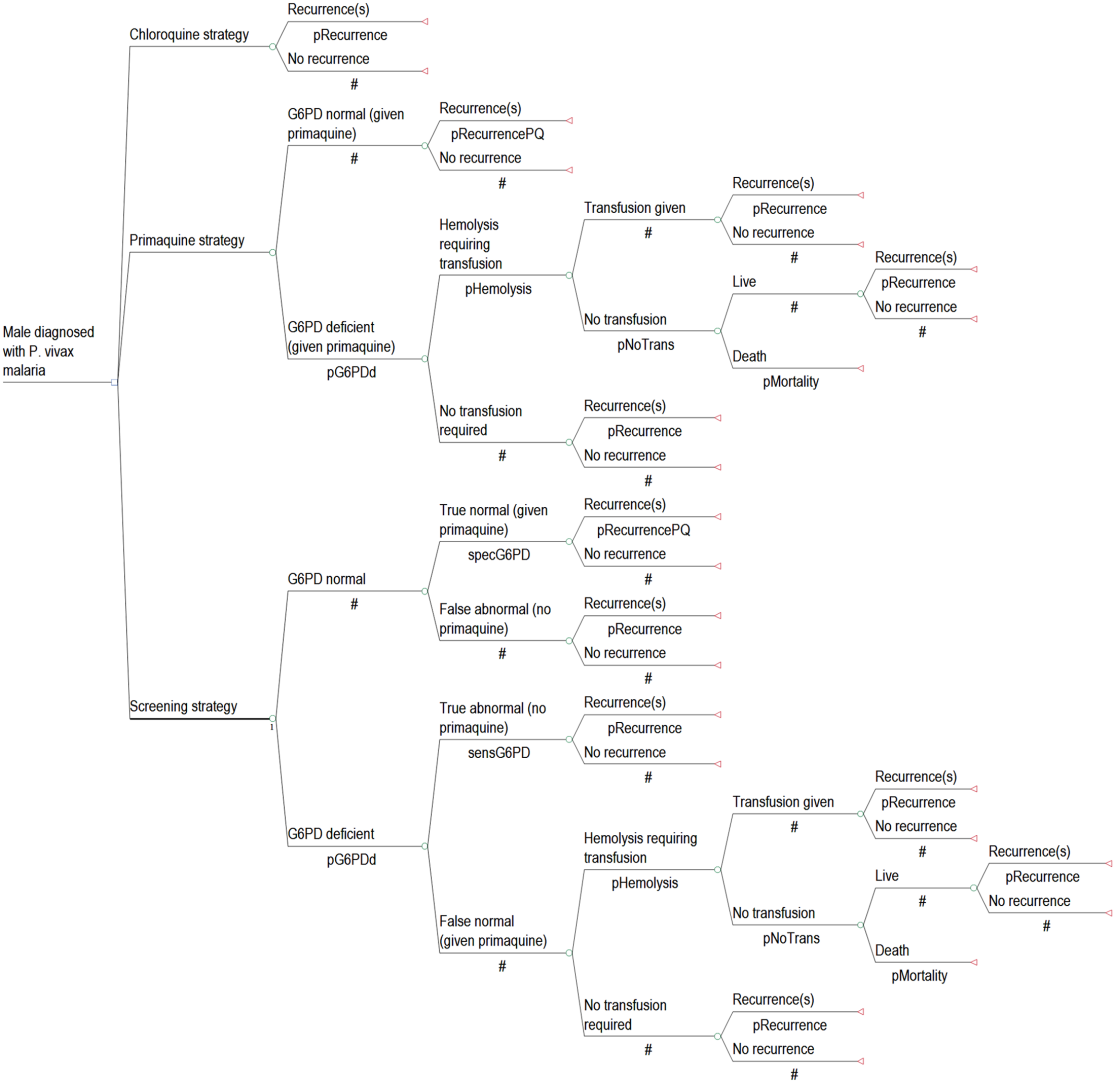
Model diagram of strategies for *P*. *vivax* treatment in males.

**Fig 2:**
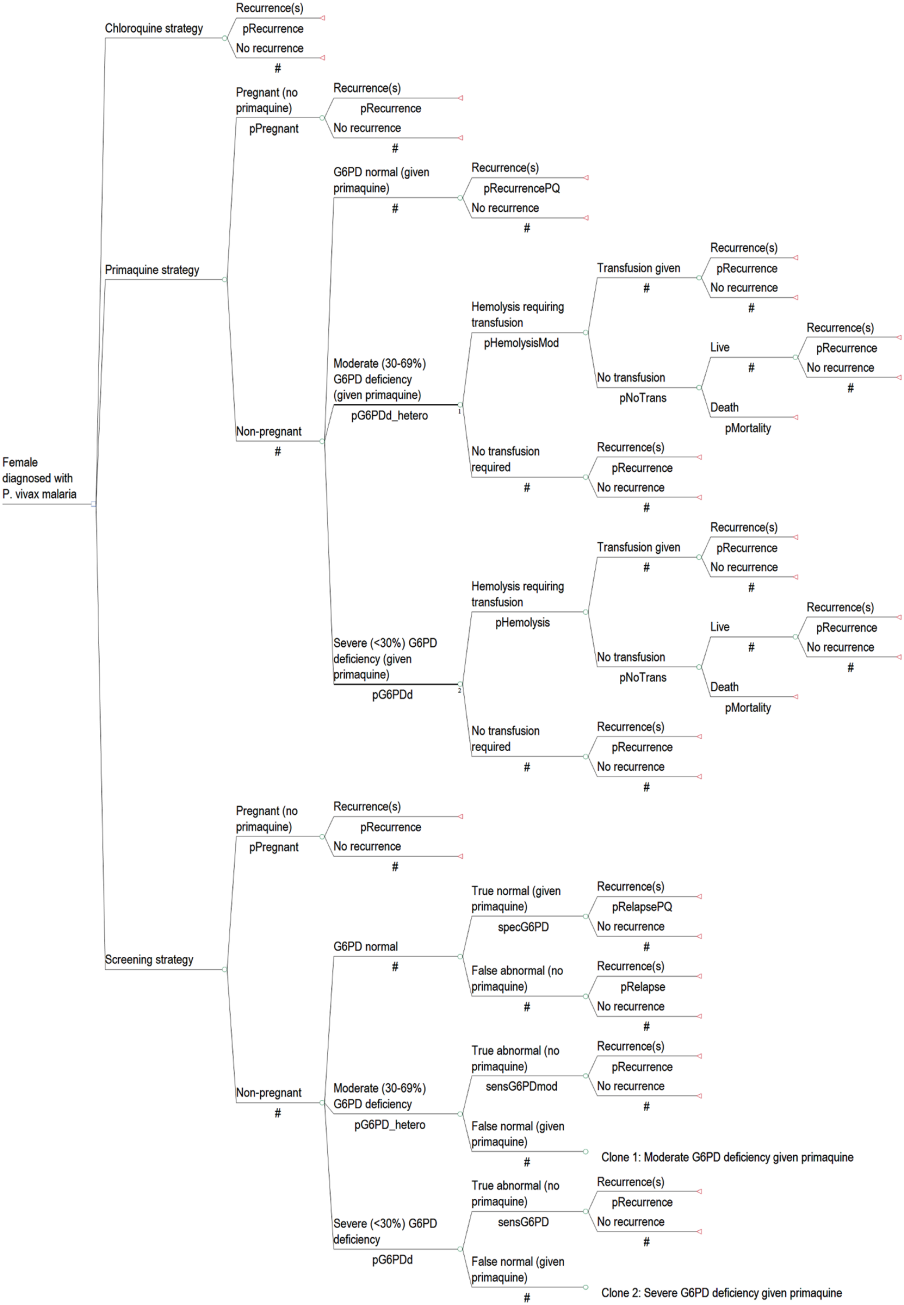
Model diagram of strategies for *P*. *vivax* treatment in females.

The analysis and results were completed separately for males (Fig 1) and females (Fig 2) to account for their differences in risks and outcomes. Firstly, as pregnant females would not be prescribed primaquine due to the unknown G6PD status of the fetus, the screening and primaquine strategies modeled the inclusion of a pregnancy test for all women of childbearing age who were unaware that they were pregnant. Those who were identified or known to be pregnant would be treated with chloroquine only and would not have a G6PD RDT. Secondly, since G6PD is an X-linked disorder, males who have deficiency are hemizygous while females can be either homozygous or heterozygous with a range of G6PD expression levels. Accordingly, G6PD deficiency was divided into two groups: severe (<30% enzyme activity) and intermediate (30–69% enzyme activity) (S2 Appendix). Generally, only females can have intermediate deficiency and the outcomes in this group were taken from heterozygotes. The G6PD RDT with a cut off of 30% activity does not detect heterozygous females with intermediate activity [16, 18]; accordingly, some women with intermediate deficiency who are identified as G6PD normal with currently available RDTs could be at risk of severe hemolysis when prescribed hemolytic drugs.

Table 2 shows the parameters used in the model. Both the screening and primaquine strategies include the cost of supervised therapy in order to reflect the additional costs required for the gains in effectiveness seen in the clinical trial. For the screening strategy, weekly supervised primaquine therapy for 8 weeks was given to those who tested G6PD abnormal [26] and the effectiveness was taken from the trial results for 14 day therapy (S1 Appendix). For each recurrence, the cost and DALY value used were taken from clinical episodes, severe malaria episodes and episodes that resulted in death and weighted proportionally. The probability for severe *P*. *vivax* was taken from a meta analysis of clinical studies using those with severe anemia, but other symptoms due to severe *P*. *vivax* were not included [2].

**Table 2:** Model parameters and sources. All costs are listed in 2014 United States Dollars.

	Base case (low, high)	Distribution (Parameters*)	Sources
Proportion of males with severe G6PD deficiency	0.137 ± 50%	Beta (13.50, 85.05)	[24]
Proportion of females with severe G6PD deficiency	0.050 ± 50%	Beta (15.17, 288.18)	Females with <30% activity (S2 Appendix)
Proportion of females with intermediate G6PD activity	0.158 ± 50%	Beta (12.96, 69.04)	Females with 30–69% activity (S2 Appendix)
Sensitivity of CareStart G6PD RDT for severe deficiency	0.99 (0.9 ‒ 1.00)	Beta (9.39, 0.09)	[18] (Low range from [16])
Sensitivity of CareStart G6PD RDT for intermediate deficiency	0.44 ± 50%	Beta (7.97, 10.14)	[18]
Specificity of CareStart G6PD RDT for males	0.99 (0.75 ‒ 1.00)	Beta (3.01, 0.03)	[18] (Low range from [16])
Specificity of CareStart G6PD RDT for females	0.97 (0.68 to 0.99)	Beta (2.59, 0.08)	[18] (Low range from [16])
Proportion who have at least one recurrence in the following year if treated with CQ	0.732	—	[23] (S1 Appendix)
Relative risk of having at least one recurrence in the following year if treated with CQ + PQ	0.219 (0.158 ‒ 1)	Lognormal (-1.52, 0.47)	[23] (S1 Appendix)
Mean number of recurrences in those treated with CQ who have at least 1 recurrence	3.537 (1 ‒ 8)	Bootstrapped data	[23] Each PSA iteration is the mean of 100 bootstrapped data points (S1 Appendix).
Mean number of recurrences in those treated with CQ + PQ who have at least 1 recurrence	1.156 (1 ‒ 2)	Bootstrapped data	[23] Each PSA iteration is the mean of 100 bootstrapped data points (S1 Appendix).
Proportion who need a blood transfusion after taking primaquine if have severe G6PD deficiency	0.109 (0.007 ‒ 0.15)	Beta (10.40, 85.01)	[27]
Proportion who need a blood transfusion after taking primaquine if have intermediate G6PD deficiency	0.005 (0.001 ‒ 0.05)	Beta (0.09, 18.72)	[27, 28] Results for female heterozygotes
Proportion who need a transfusion due to hemolysis but do not receive it	0.100 (0.010 ‒ 0.150)	Beta (8.77, 78.90)	Assumption for both severe and intermediate deficiency
Mortality due to not receiving a needed transfusion	0.100 (0.010 ‒ 0.500)	Beta (2.22, 19.95)	Assumption for both severe and intermediate deficiency
Proportion of females who are pregnant	0.11 ± 50%	Beta (14.05, 113.65)	Assumption
Proportion of females who need to take a pregnancy test	0.50 ± 50%	Beta (6.92, 6.92)	Assumption. All women of childbearing age are tested unless they disclose pregnancy
**COSTS**			
CareStart G6PD RDT	1.75 (0.88 ‒ 10.00)	Gamma (0.38, 4.64)	RDT (1.50) [29] and administration (0.25) [30]
Primaquine pill	0.06 ± 50%	Gamma (24.71, 0.00)	[31] 14 days of 30mg/day; 8 weeks of 45mg/week. Assumed weight = 60kg.
One session of supervised primaquine therapy by a community health worker	1.67 ± 50%	Gamma (16.15, 0.10)	[32] One-half day of work for a medium accessible area with no incentives per supervision
Initial episode and clinical *P*. *vivax* recurrence	7.86 ± 50%	Gamma (16.10, 0.49)	[33] Outpatient visit, excluding screening and treatment costs
Severe *P*. *vivax* recurrence	196.22 (95% CI: 135.08 ‒ 271.39)	Gamma (31.48, 6.23)	[34] Inpatient visit for severe *Plasmodium falciparum*
Hemolytic episode requiring transfusion	320.71 (166.03 ‒ 401.53)	Gamma (32.55, 9.85)	One unit of blood (25.31) [35] and 7 day inpatient stay (295.40) [33]
Pregnancy test	0.10 ± 50%	Gamma (16.10, 0.01)	SMRU clinic records
**DALYS**			
Proportion of *P*. *vivax* cases that are severe	0.020 (95% CI: 0.013 ‒ 0.027)	Beta (31.41, 1539.05)	[2]
*P*. *vivax* mortality	0.0001 (95% CI: 0–0.001)	Beta (0.09, 929.01)	Assumption
Life expectancy for males aged 20–24 in Myanmar	48.9 ± 20%	Gamma (96.79, 0.51)	[36]
Life expectancy for females aged 20–24 in Myanmar	52 ± 20%	Gamma (96.79, 0.54)	[36]
Length disability: clinical malaria	0.008 (0.003 ‒ 0.019)	Beta (3.27, 404.86)	3 days (range 1–7)
Length disability: moderate anemia due to clinical malaria	0.083 (0.04 ‒ 0.17)	Beta (5.39, 59.33)	[37] 1 month (range 0.5 to 2)
Length disability: severe malaria	0.019 (0.008 ‒ 0.027)	Beta (16.91, 872.94)	7 days (range 3–10)
Length disability: severe anemia due to severe malaria or hemolysis	0.250 (0.083 ‒ 0.500)	Beta (3.59, 10.76)	3 months (range 1–6)
Disability weight for clinical malaria	0.053 (0.033 ‒ 0.081)	Gamma (18.12, 0.003)	[38] infectious disease: moderate acute episode
Disability weight for moderate anemia due to clinical malaria	0.058 (0.038 ‒ 0.086)	Gamma(21.70, 0.003)	[38]
Disability weight for severe malaria	0.210 (0.139 ‒ 0.298)	Gamma (26.50, 0.008)	[38] infectious disease: severe acute episode
Disability weight for severe anemia due to severe malaria or hemolysis	0.164 (0.112 ‒ 0.228)	Gamma (30.37, 0.005)	[38]

* Parameters: Beta (alpha, beta), Lognormal (log (mean), log (standard deviation)) and Gamma (shape, scale).

The probability of having a hemolytic episode that requires a transfusion in individuals with severe and intermediate G6PD deficiency treated with primaquine was taken from a study of children treated with Dapsone in Africa [27]. While this population may be different in terms of age and G6PD variant from those being treated with primaquine on the northwestern border of Thailand with Myanmar, this was the best available data on transfusion risk. The probability of hemolysis requiring transfusion for severe G6PD deficiency was taken from the proportion of hemizygotes and homozygotes in the study while the probability for females with intermediate deficiency was taken from the proportion of heterozygotes. It was assumed that 10% of patients requiring a transfusion did not receive one; of those, 10% died as a result of not receiving a transfusion. It was assumed that the decision to give a transfusion was made by a physician and that the costs are included in the cost of transfusion.

Costs of commodities and service delivery were taken from Myanmar and Thailand and supplemented by international sources when needed. Costs are reported in 2014 United States Dollars (US$). The cost of supervised therapy was taken from data on annual costs of a community health worker in Myanmar [32], assuming one half-day of pay per observation. The cost of hospitalization for a blood transfusion was included for severe hemolytic episodes which did not lead to death. Table 1 describes the costs for recurrences.

The DALY weights were taken from the 2010 Global Burden of Disease Study [38]. These weights were combined with life tables for Myanmar [36] and assumptions about the length of illness to calculate the DALY burden for each strategy.

In instances where the screening strategy averted DALYs while costing more money the incremental cost-effectiveness ratios (ICER) was calculated:

$$ICER\;=\;\frac{Cost_{s}–\;Cost_{b}}{DALY_{b}-DALY_{s}}$$

Where *Cost* is the total cost of the strategy and *DALYs* is the total DALYs of the corresponding strategy. While the gross domestic product per capita for Myanmar is approximately US$1200 [39], it has been argued that a lower willingness to pay threshold may be appropriate lower income countries [40]; consequently, a threshold of US$500 was chosen to reflect the resource limitations of healthcare facilities serving migrant and refugee communities.

### Sensitivity analyses

A one-way sensitivity analysis was conducted to examine the impact of parameter values on the overall outcome. Low and high values were taken from 95% confidence intervals (CIs) when available. When not available, the point estimate was varied by 50% and given wider intervals when necessary to reflect the uncertainty (Table 2). Results that varied from the base case by more than US$0.05 or 0.0002 DALYs averted were reported.

A probabilistic sensitivity analysis (PSA) was conducted to incorporate the uncertainty of all parameters over 1000 sampling iterations using the parameter ranges used in the one-way sensitivity analysis. Table 2 lists the distributions used in the PSA. The sum of squared differences was minimized from the specified ranges to produce the shape values for the beta and gamma distributions and random numbers were generated from these distributions. The mean number of recurrences for each iteration was calculated from 100 bootstrapped data points that were randomly sampled from the data set with replacement (S1 Appendix). The PSA produced a mean estimate and 95% credible intervals (CrIs) for the costs, DALYs and incremental results.

A key concern is adherence to primaquine regimens by the patients as well as compliance to guidelines by prescribers, which is collectively referred to as “adherence” here. In order to account for this, a two-way sensitivity analysis examined the interplay of costs and benefits depending on adherence to the primaquine strategy (whether primaquine was administered to the patient and the full course taken) and screening strategy (whether a G6PD RDT plus primaquine was administered to the patient and the full course taken). This cohort analysis assumed that at 0% adherence all individuals have a relative risk and mean number of recurrences equivalent to receiving chloroquine only. The proportion of individuals in the population who are adherent increases steadily until 100% adherence, which assumes that recurrences are equivalent to the base case. Costs of supervised primaquine and G6PD screening were also varied accordingly. Assumptions about adherence in individuals with G6PD deficiency who receive 14 day primaquine remain the same as the base case analysis.

## Results

### Comparison with the chloroquine strategy

Costs and DALYs for each strategy are shown in Table 3 and the cohort results are in Table 4. On the Thailand-Myanmar border, the screening strategy averted more DALYs than the chloroquine strategy: 0.026 for males and 0.024 for females. These gains were produced for similar costs. The base case ICERs were US$6.3 and US$11.7 per DALY averted for males and females respectively.

**Table 3:** Costs and disability adjusted life year (DALY) results over one year per primary episode treated in the base case analysis and probabilistic sensitivity analysis (PSA). All costs are in 2014 United States Dollars.

	Mean costs		Mean DALYs	
	Base case	PSA (95% CrI*)	Base case	PSA (95% CrI*)
Males				
Chloroquine strategy	38.0	37.8 (24.6 to 55.5)	0.034	0.034 (0.009 to 0.137)
Primaquine strategy	45.3	45.8 (31.6 to 62.6)	0.019	0.019 (0.007 to 0.050)
Screening strategy	38.2	38.6 (25.2 to 55.3)	0.007	0.008 (0.003 to 0.018)
Females				
Chloroquine strategy	38.0	38.3 (24.0 to 54.4)	0.034	0.034 (0.009 to 0.155)
Primaquine strategy	40.5	41.2 (28.3 to 59.0)	0.015	0.015 (0.006 to 0.040)
Screening strategy	38.3	39.1 (25.6 to 57.3)	0.011	0.011 (0.004 to 0.030)

* Credible interval.

**Table 4:** The table shows simulated outcomes for 1000 *P*. *vivax* malaria patients of undetermined G6PD status at attendance, if they were managed according to each of three strategies (chloroquine, primaquine and screening) according to G6PD status, treatment given and the test result if screened.

Initial treatment by G6PD status	Males			Females		
	Strategy			Strategy		
	Chloroquine	Primaquine	Screening	Chloroquine	Primaquine	Screening
G6PD normal who get primaquine and have no recurrences	—	725	718	—	592	592
G6PD normal who get primaquine and have at least one recurrence	—	138	137	—	113	113
G6PD normal who get chloroquine only and have no recurrences	231	—	2	212	—	—
G6PD normal who get chloroquine only and have at least one recurrence	632	—	6	580	—	—
G6PD deficients* who get chloroquine only	137	—	—	208	—	—
G6PD deficients* who get weekly primaquine	—	—	136	—	—	106
Severe G6PD deficients who get 14 day primaquine, do not complete treatment but do not require a transfusion	—	122	1	—	40	0.40
Intermediate G6PD deficients who get 14 day primaquine and complete treatment	—	—	—	—	140	78
G6PD deficients* who get primaquine, hemolyze and receive a required transfusion	—	13	0.13	—	5	0.40
G6PD deficients* who get primaquine, hemolyze, do not receive a required transfusion and live	—	1	0.01	—	0.50	0.04
G6PD deficients* who get primaquine, hemolyze and die due to not receiving a transfusion	—	0.15	0.001	—	0.06	0.004
Do not receive primaquine due to pregnancy	—	—	—	—	110	110

* For women, this includes both severe and intermediate G6PD deficiency.

**Fig 3:**
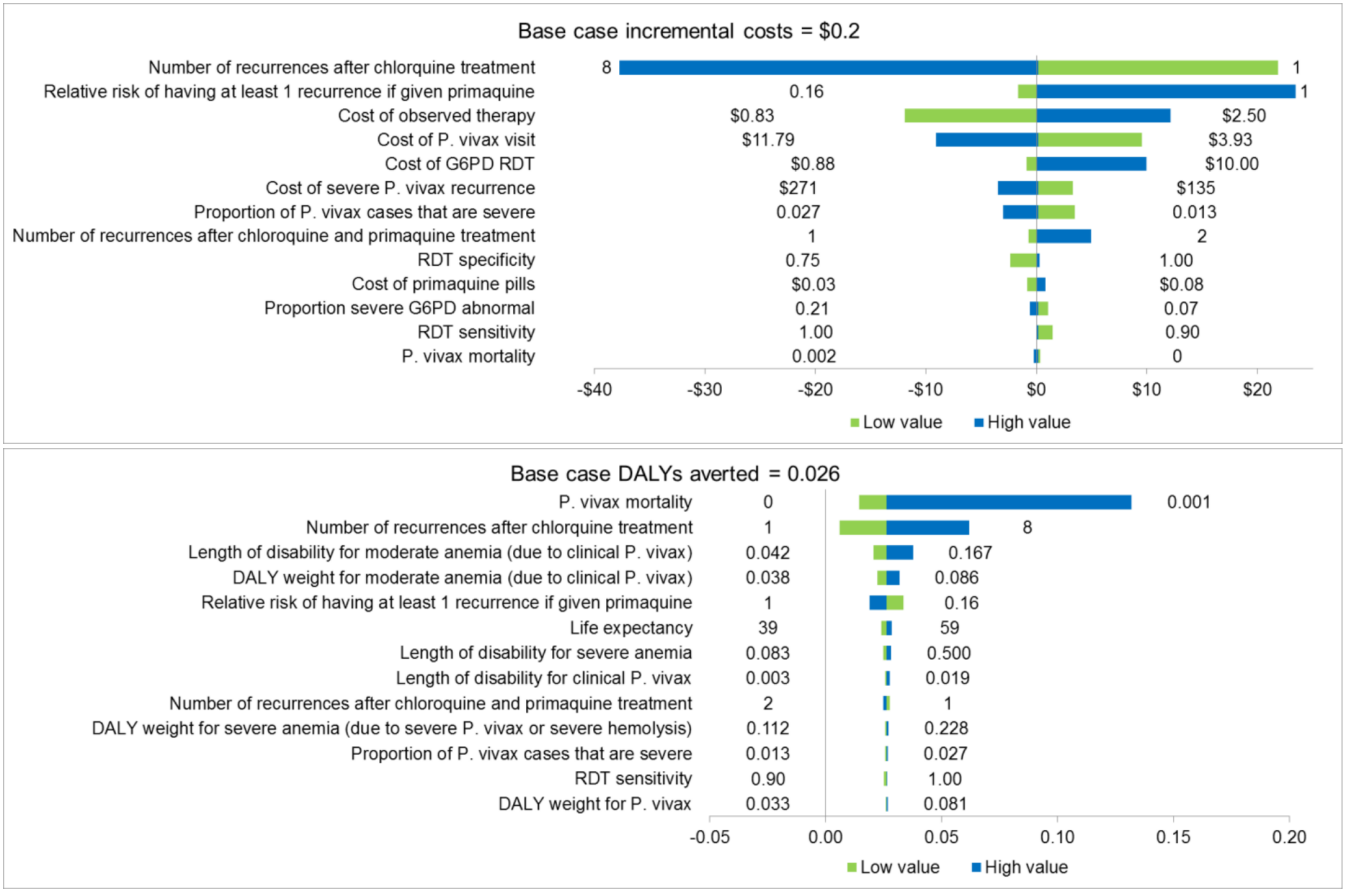
One-way sensitivity analysis results on costs and disability-adjusted life-years (DALYs) for the comparison of the screening strategy with the chloroquine strategy in males. The ranges used are specified in Table 2. See S3 Appendix for all one-way sensitivity analysis results.

Fig 3 shows the results of the one-way sensitivity analysis in males (see S3 Appendix for all results). The screening strategy always averted more DALYs than the chloroquine strategy (S3C and S3D Appendix). Costs for the screening strategy were highest when radical cure had a low impact on recurrences, when the costs of supervised therapy and the G6PD RDT were increased, and also when the cost of a recurrence was decreased (S3A and S3B Appendix). The only assumptions that made the screening strategy cost over US$500 per DALY averted were lowering the number of recurrences after chloroquine to 1 (US$3678.7 in males and US$3724.5 in females) and assuming the same relative risk of having at least one recurrence in females (US$1223.6).

**Fig 4:**
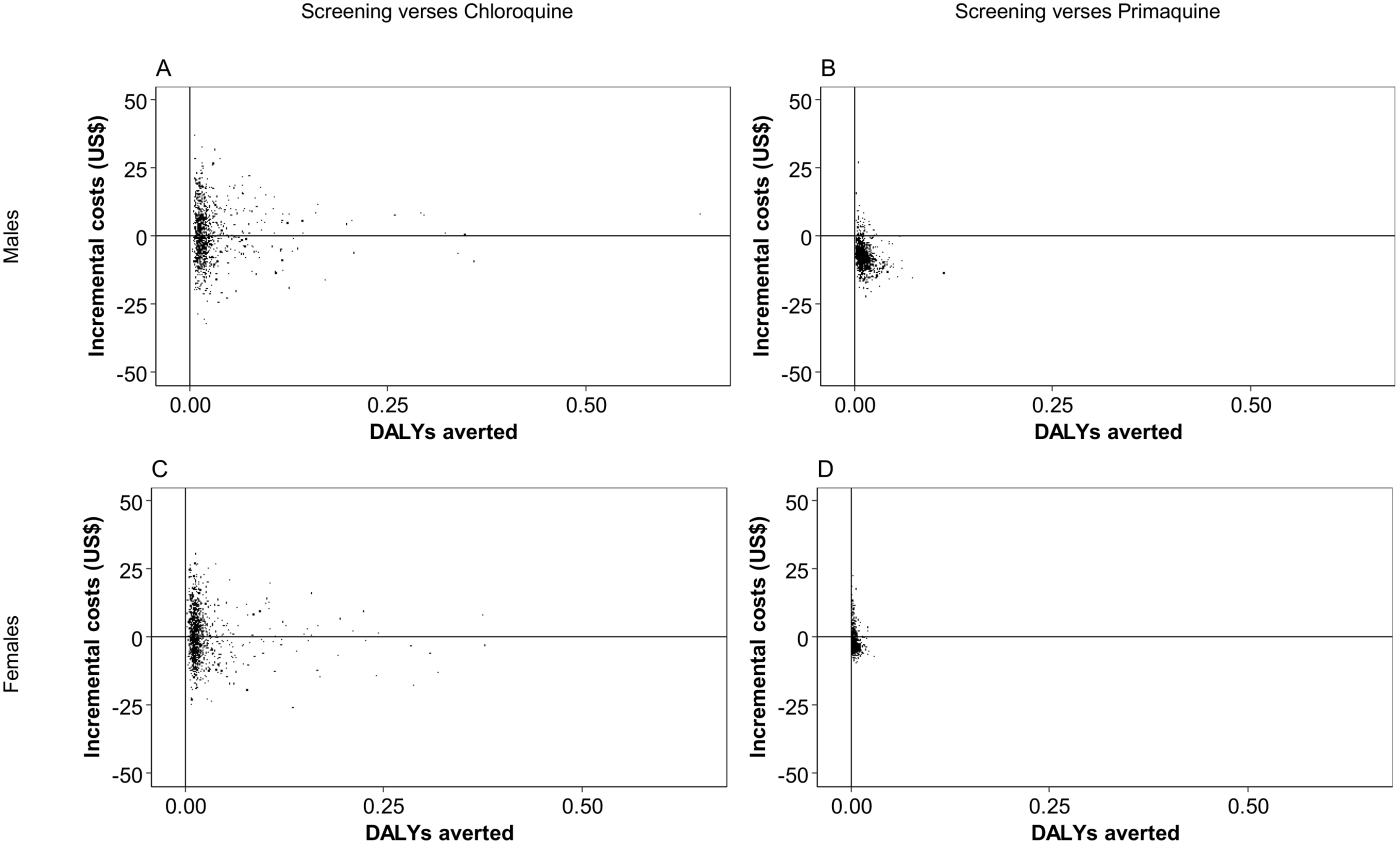
Cost-effectiveness plane showing the incremental costs and DALYs averted from the probabilistic sensitivity analysis. Results show the screening strategy versus: A) the chloroquine strategy in males, B) the primaquine strategy for males, C) the chloroquine strategy in females and D) the primaquine strategy in females.

**Fig 5:**
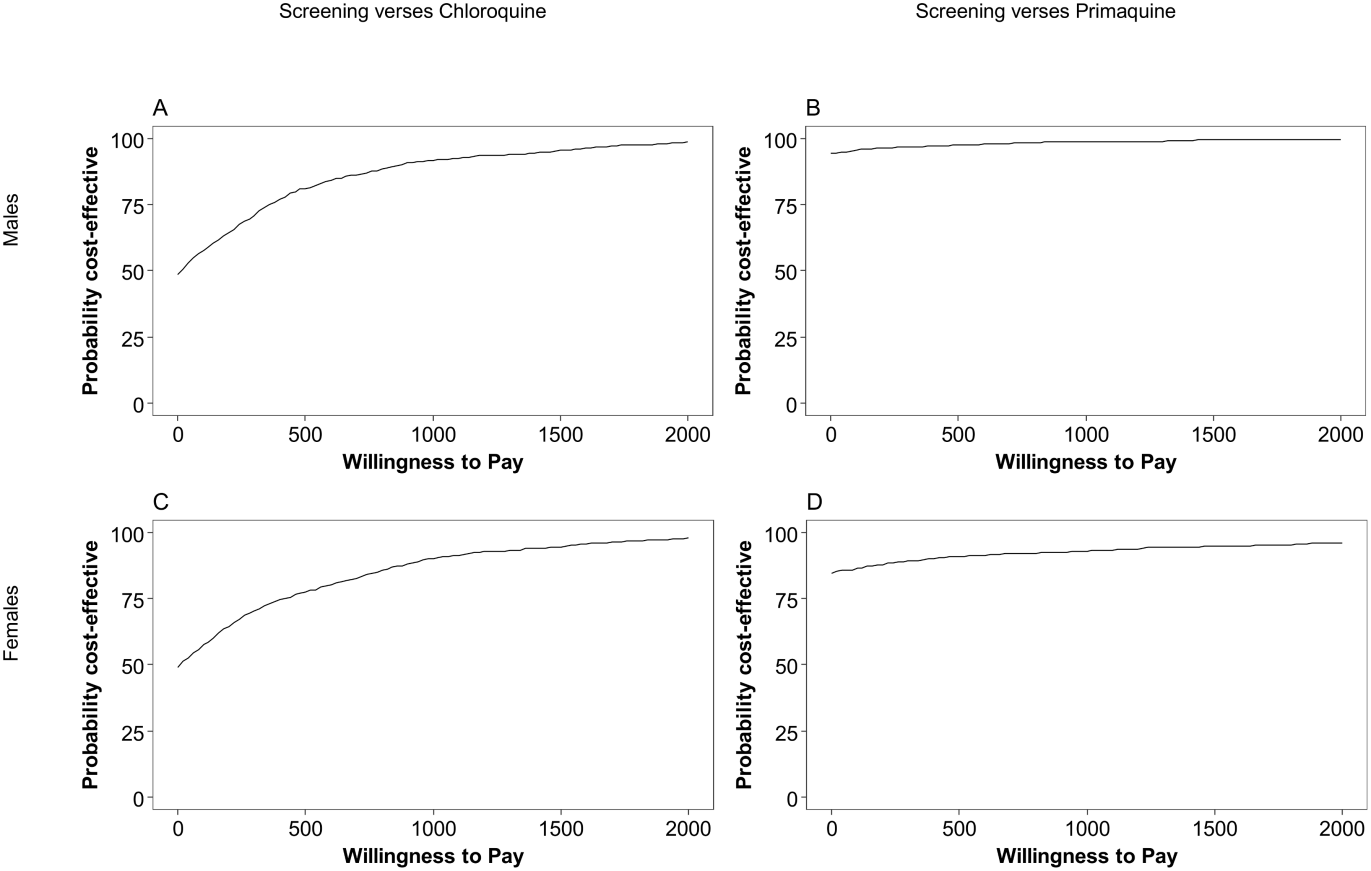
Cost-effectiveness acceptability curves. Results show the screening strategy versus: A) the chloroquine strategy in males, B) the primaquine strategy for males, C) the chloroquine strategy in females and D) the primaquine strategy in females.

The mean costs and DALYs and CrIs estimated by the PSA are shown in Table 4. The screening strategy costing more than the chloroquine strategy with mean incremental cost of US$0.8 (95%CrI: –17.4 to 19.7) and 0.026 DALYs averted (95%CrI: 0.007 to 0.117) per male (Fig 4A). At a willingness to pay threshold of US$500, the screening strategy had an 81.2% probability of being cost-effective (Fig 5A). The PSA resulted in a mean incremental cost of US$0.75 (95%CrI: –15.0 to 20.0) with 0.023 DALYs averted (95%CrI: 0.006 to 0.122) per female (Fig 4C) and a 77.6% probability of being cost-effective at a willingness to pay threshold of US$500 (Fig 5C). The ICERs were US$31.3 per DALY averted for males and US$32.4 for females.

### Comparison with the primaquine strategy

Again, the screening strategy resulted in better health outcomes in the base case with 0.011 DALYs averted in males and 0.004 in females (Table 3). The health gains in females were more modest due to their overall lower probability of hemolysis requiring transfusion. In addition, the screening strategy produced cost savings of US$7.1 and US$2.2 per male and female initially treated, respectively (Table 3). The simulation output indicated that one death due to hemolysis would be expected for every 6682 males and 15,994 non-pregnant females treated using the primaquine strategy. This would be reduced to one death per 668,164 males and 201,198 non-pregnant females treated with the screening strategy (Table 4).

The one-way sensitivity analysis showed that changes in the parameter values had a smaller impact on the costs when comparing screening and primaquine strategies, especially for females, and results that consistently averted DALYs (S3E–S3H Appendix). The parameters related to mortality, the need for transfusion and the prevalence of G6PD deficiency having the highest impact on DALY results (S3G and S3H Appendix). The screening strategy was cost saving with the exception of raising the G6PD RDT cost to US$10.0, which caused an incremental cost for the screening strategy of US$235.9 and US$1602.1 for males and females, respectively (S3E and S3F Appendix). The screening strategy remained cost saving even at low levels of G6PD deficiency (7%).

The PSA showed a mean cost savings of US$7.3 (95%CrI: -15.4 to 3.4) and 0.012 DALYs averted (95%CrI: 0.001 to 0.113) in males (Fig 4B), and a mean cost savings of US$2.2 (95%CrI: –6.2 to 6.7) and 0.004 DALYs averted (95%CrI: 0.000 to 0.029) in females (Fig 4D). The screening strategy had a 97.7% probability of being cost-effective for males at a willingness to pay threshold of US$500.0 (Fig 5B). For females, the probability was 91.1% (Fig 5D). Table 3 shows the cost and DALY estimates from the PSA.

**Fig 6:**
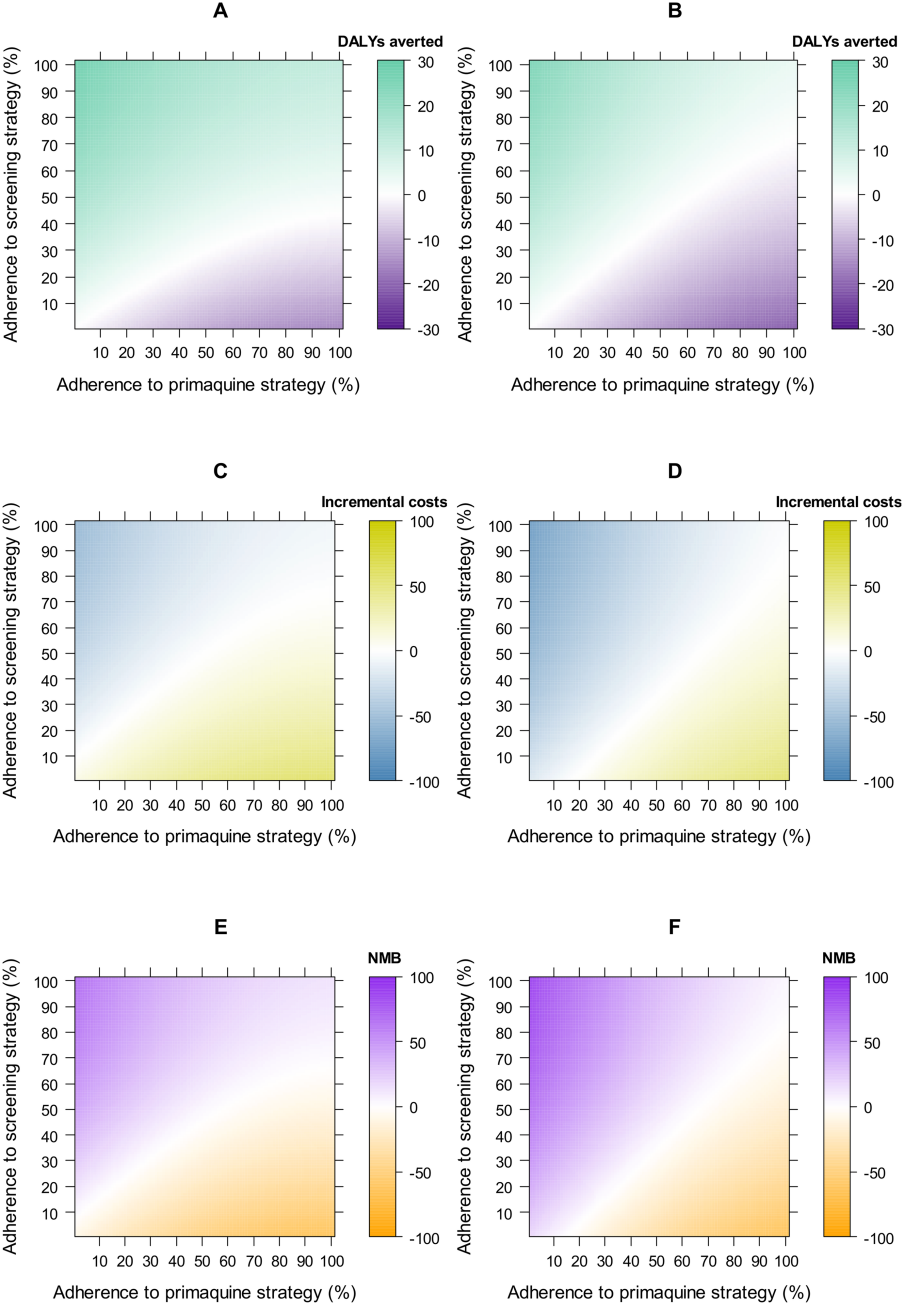
Two-way sensitivity analysis results showing the impact of changes in the level of adherence to primaquine regimens. Green represents disability-adjusted life-years (DALYs) averted by the screening strategy for a cohort of 1000 individuals. For costs, yellow indicates increased costs and blue indicates cost savings for the screening strategy for one individual. For net monetary benefit (*NMB*), purple indicates scenarios where the screening strategy would be cost-effective at a threshold of US$500 (*T*) where *NMB = T × ΔDALYs– ΔCosts*.

The two-way analysis (Fig 6) demonstrated that the screening strategy would be cost-effective in scenarios where it is used to maintain or increase the number of patients who are adherent to their primaquine regimens. The impact of switching to the screening strategy was slightly less in females due to the exclusion of pregnant women from primaquine treatment and the low sensitivity of the G6PD RDT in women with intermediate G6PD deficiency.

### Web-based application

Due to the extensive heterogeneity and parameter uncertainty around key parameter estimates, notably relapse patterns [25], G6PD variants and prevalence [10] and costs, a web-based interface was built using the R-Shiny application so that the model could be adapted to other settings as need be. See website (https://malaria.shinyapps.io/g6pd_screening/).

## Discussion

Point of care G6PD RDTs offer the opportunity for the safe uptake of primaquine for the prevention of recurrences. Our findings suggest that on the Thailand-Myanmar border the use of G6PD RDTs to identify patients with G6PD deficiency before supervised primaquine is likely to provide significant health benefits (equivalent to between 1 and 9 days of perfect health) compared to giving chloroquine alone or giving 14 day primaquine without G6PD testing. Furthermore, the use of point of care G6PD RDTs will potentially save costs or, at most, increase them moderately.

Primaquine is currently the only licensed hypnozonticidal drug, but healthcare professionals who treat *P*. *vivax* cases are often more concerned with avoiding the immediate risk of hemolysis than with protecting the patient from the risks associated with future relapses. In other settings, primaquine may be administered without G6PD testing, putting individuals with G6PD deficiency at risk of severe hemolysis, although the degree of risk will depend upon local G6PD variants and their prevalence. In the scenario presented, the use of G6PD RDTs will save costs while averting DALYs compared to a policy in which primaquine is administered without G6PD testing. While our results give a high probability of cost savings when switching from the primaquine strategy to the screening strategy, this should not deter radical cure without screening in settings where screening is unavailable as the primaquine strategy averted more DALYs than the chloroquine strategy.

Our model is based on supervised primaquine therapy and hence our findings may not be applicable to other settings where unsupervised primaquine is the norm and adherence to a complete course of treatment and thus effectiveness may be low [41]. Our two-way analysis on adherence (Fig 6) enabled comparison between settings with varying adherence and how this impacts upon cost effectiveness. The screening strategy averts more DALYs than the primaquine strategy, even at relatively high primaquine strategy adherence and low screening strategy adherence. Shorter drug courses, such as 7 day primaquine and tafenoquine, should contribute to higher adherence levels and reduced costs for the primaquine and screening strategies.

Overall, the screening strategy was less cost-effective in women as compared to men. This reflects a greater proportion of women who are excluded from receiving primaquine due to pregnancy and the lower risk of severe hemolysis in females with intermediate G6PD deficiency. Since men represented 65% of patients in the trial that the recurrence data were derived from (S1 Appendix), the overall cost-effectiveness estimates per person presenting with *P*. *vivax* malaria would likely be closer to the results for males.

The cost-effectiveness of using G6PD RDTs is also dependent on the diagnostic accuracy of the test. Our model draws on studies conducted on the Thailand-Myanmar border, which demonstrated a high sensitivity in healthy volunteers. While other studies have shown similar results [16, 17] in healthy volunteers, a recent study in Brazil found that the sensitivity of the CareStart G6PD RDT dropped to 50% in patients with malaria compared to 80% in those who did not [15]. A recent cost-effectiveness analysis of male patients with *P*. *vivax* malaria in Brazil used a low sensitivity for the CareStart G6PD RDT (46%) but still found it to be more cost-effective than both the BinaxNOW test and routine care; where the analysis also involved the prescription of primaquine without having a G6PD test [42]. This study, however, used the endpoints ‘adequately diagnosed case’ and ‘hospitalization avoided’ instead of DALYs. The Brazilian population was given a 94% probability of hospitalization when primaquine was given to G6PD deficient men. Our model differs in that we assume a lower rate of hospitalization due to severe hemolysis. We also include results for both genders and report DALYs, enabling comparisons with interventions for other diseases.

Our study has a number of limitations, mostly related to our model assumptions. The cost-effectiveness of the screening strategy would be increased if it included the onward transmission of *P*. *vivax* or the longer term impact of repeated episodes, such as anemia, malnutrition and all-cause mortality. This is particularly relevant in areas such as the Thailand-Myanmar border where the estimated proportion of recurrences due to relapses is estimated to be 78%. The cost-effectiveness may decrease if some individuals were not able to metabolize primaquine, if healthcare workers were not able to utilize G6PD RDTs or supervise primaquine regimens, if the prevalence of G6PD deficiency in those presenting with *P*. *vivax* was lower to that in the general population, if the diagnostic accuracy of the G6PD test were lower, if healthcare facilities providing care for hemolytic episodes were not accessible or if the operational costs of implementing a switch to the screening strategy were included. These parameter limitations are similar to those highlighted in a recent review of the costs and cost-effectiveness of *P*. *vivax* control and elimination [43].

Finally, our model is limited by the paucity of data available in the literature for some parameter values, including the mortality rate for those who have a primaquine-induced hemolytic episode requiring transfusion but do not receive them. Our assumptions of primaquine induced mortality were derived from previous risks of mortality in patients treated with Dapsone in Africa and equated to a population risk of 1 in 6,682 administrations to males and 1 in 15,994 administrations in females. These risks are significantly higher than the risks documented in a previous review [11] but the screening strategy averted more DALYs than the primaquine strategy at a lower level of primaquine-induced mortality, though this is likely due to the utilization of weekly primaquine by the screening strategy. Other variables, such as the prevalence of G6PD deficiency will vary greatly depending on the epidemiological setting. Whilst it would be beneficial to gather more robust parameter estimates on which to base informed policy decisions, this should be tempered by the feasibility of gathering such data and the potential benefits of implementing appropriate policies sooner, especially in the context of elimination.

Although our model is relatively simple, it provides a useful starting point for policy makers to compare the risks and benefits of using G6PD RDTs to enable the safe and effective use of primaquine. To assist in this process we provide an online tool with which policy makers and healthcare providers can vary the assumptions made in the model in keeping with local scenarios and as additional data becomes available (https://malaria.shinyapps.io/g6pd_screening/). As the only licensed antimalarial for the radical cure of *P*. *vivax* infections, primaquine will be a critical tool for the elimination of all malaria [44] and for the health gains provided to patients. The currently available G6PD RDTs can identify G6PD deficient males, making the screening strategy an attractive option regardless of current practice. In situations where blood transfusions are not accessible, further information may be required on the prevalence of G6PD deficiency and associated risk of hemolysis in females with intermediate G6PD deficiency who test normal by current G6PD RDT methods [28]. Despite the initial cost, point of care RDTs avert DALYs by reducing recurrences while diminishing the hemolytic risk in G6PD deficient patients.

## Supporting information

S1 Appendix *P*. *vivax* recurrence data. (XLSX)

S2 Appendix Study description for determining G6PD activity in females. (PDF)

S3 Appendix One-way sensitivity analysis results on costs and disability-adjusted life-years (DALYs). (PDF)
