# Supplementary material for: Using G6PD tests to enable the safe treatment of Plasmodium vivax infections with primaquine on the Thailand-Myanmar border: A cost-effectiveness analysis
Source: PLoS Negl Trop Dis. 2017 May 24;11(5):e0005602. doi: 10.1371/journal.pntd.0005602 (PMC5460895; doi:10.1371/journal.pntd.0005602)
Supplement: S2 Appendix — (PDF) [file pntd.0005602.s002.pdf]

Study population and samples

Healthy women were enrolled over a four month time period as part of an ongoing project by the Shoklo Malaria Research Unit (SMRU) among the female migrant and refugee population living at the Thailand-Myanmar border with a target enrolment of 300. No exclusion criteria specific to G6PD status were applied; therefore, the sample is believed to represent the general female population of the area. A venous blood sample was taken from each participant and transported within eight hours to the central laboratory for analyses.

Laboratory methods

Screening for glucose-6-phosphate-dehydgrogenase (G6PD) deficiency using the Fluorescent Spot Test (R&D Diagnostic, Greece). Five microliters of blood were mixed with reagents, spotted on filter paper after 10 minutes of incubation and allowed to air-dry over 20 minutes. Spots were visualized under ultraviolet light and normal or deficient phenotype assigned based on fluorescence. Quantitative phenotype was assessed by spectrophotometry according to WHO protocol on whole blood depleted of white blood cells [1]. G6PD activity was calculated after normalization with hemoglobin (Hb) concentration and expressed as percentage of the population median which was previously established using the same technique.

Statistical analysis

Three categories of G6PD activity were chosen: <30%, 30-69% and ≥70%. The percentage of women in each of those categories was calculated using R statistical software [2].

Results

322 women were enrolled in the study. Of those who were enrolled, 5.0% (16/322) had less than 30% G6PD activity and 15.8% (51/322) tested as having between 30 and 69% G6PD activity.

References

1. Beutler E, Blume KG, Kaplan JC, Lohr GW, Ramot B, Valentine WN. International Committee for Standardization in Haematology: recommended methods for red-cell enzyme analysis. British journal of haematology. 1977;35(2):331-40. Epub 1977/02/01. PubMed PMID: 857853.

2. R Core Team. R: A language and environment for statistical computing. Vienna, Austria: R Foundation for Statistical Computing; 2016.
