# Supplementary material for: Using G6PD tests to enable the safe treatment of Plasmodium vivax infections with primaquine on the Thailand-Myanmar border: A cost-effectiveness analysis
Source: PLoS Negl Trop Dis. 2017 May 24;11(5):e0005602. doi: 10.1371/journal.pntd.0005602 (PMC5460895; doi:10.1371/journal.pntd.0005602)
Supplement: S3 Appendix — (PDF) [file pntd.0005602.s003.pdf]

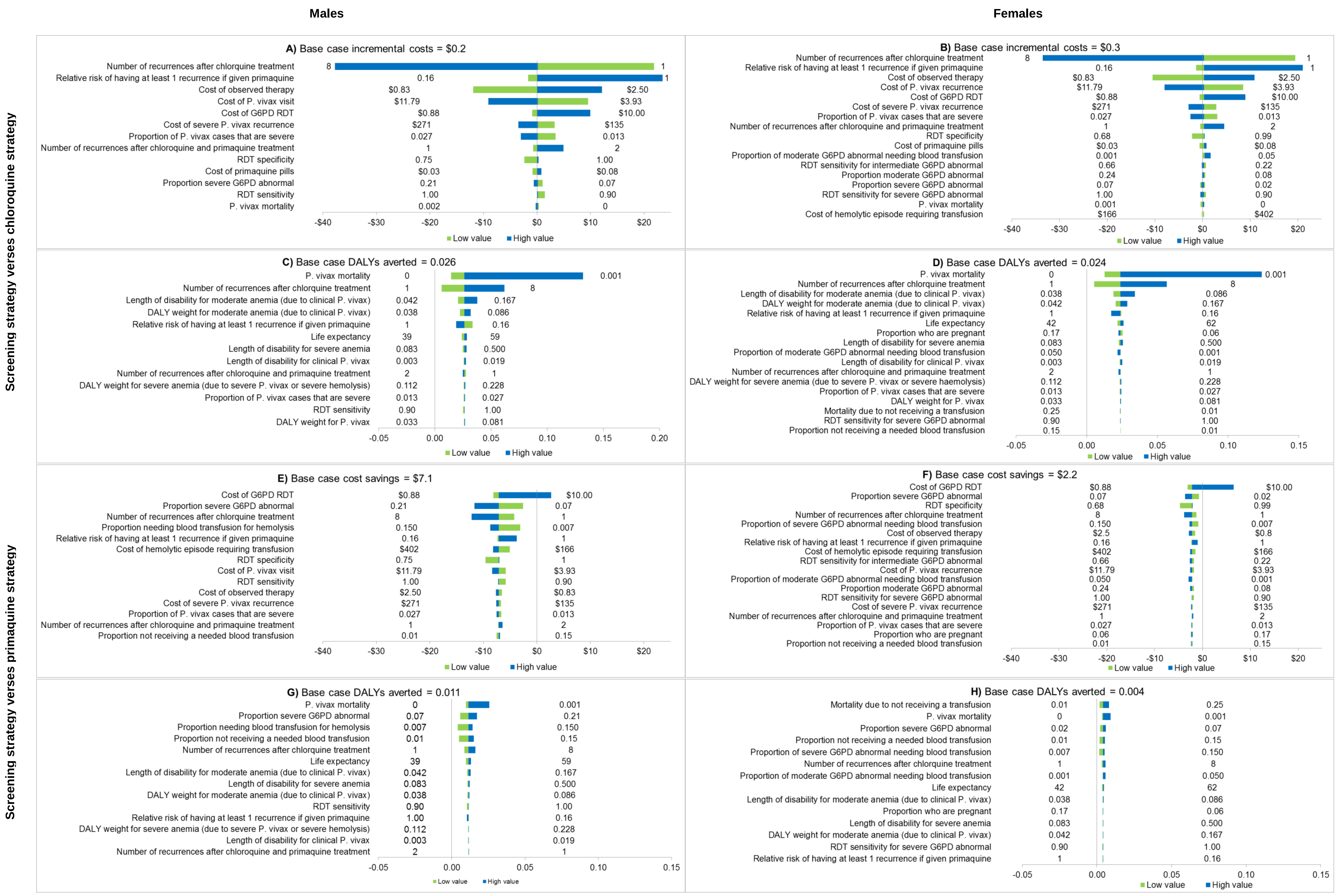
Males
Females
Screening strategy verses chloroquine strategy
Screening strategy verses primaquine strategy
A) Base case incremental costs = $0.2
Number of recurrences after chlorquine treatment 8 1
Relative risk of having at least 1 recurrence if given primaquine 0.16 1
Cost of observed therapy $0.83 $2.50
Cost of P. vivax visit $11.79 $3.93
Cost of G6PD RDT $0.88 $10.00
Cost of severe P. vivax recurrence $271 $135
Proportion of P. vivax cases that are severe 0.027 0.013
Number of recurrences after chloroquine and primaquine treatment 1 2
RDT specificity 0.75 1.00
Cost of primaquine pills $0.03 $0.08
Proportion severe G6PD abnormal 0.21 0.07
RDT sensitivity 1.00 0.90
P. vivax mortality 0.002 0
-$40 -$30 -$20 -$10 $0 $10 $20
Low value High value
B) Base case incremental costs = $0.3
Number of recurrences after chlorquine treatment 8 1
Relative risk of having at least 1 recurrence if given primaquine 0.16 1
Cost of observed therapy $0.83 $2.50
Cost of P. vivax recurrence $11.79 $3.93
Cost of G6PD RDT $0.88 $10.00
Cost of severe P. vivax recurrence $271 $135
Proportion of P. vivax cases that are severe 0.027 0.013
Number of recurrences after chloroquine and primaquine treatment 1 2
RDT specificity 0.68 0.99
Cost of primaquine pills $0.03 $0.08
Proportion of moderate G6PD abnormal needing blood transfusion 0.001 0.05
RDT sensitivity for intermediate G6PD abnormal 0.66 0.22
Proportion moderate G6PD abnormal 0.24 0.08
Proportion severe G6PD abnormal 0.07 0.02
RDT sensitivity for severe G6PD abnormal 1.00 0.90
P. vivax mortality 0.001 0
Cost of hemolytic episode requiring transfusion $166 $402
-$40 -$30 -$20 -$10 $0 $10 $20
Low value High value
C) Base case DALYs averted = 0.026
P. vivax mortality 0 0.001
Number of recurrences after chlorquine treatment 1 8
Length of disability for moderate anemia (due to clinical P. vivax) 0.042 0.167
DALY weight for moderate anemia (due to clinical P. vivax) 0.038 0.086
Relative risk of having at least 1 recurrence if given primaquine 1 0.16
Life expectancy 39 59
Length of disability for severe anemia 0.083 0.500
Length of disability for clinical P. vivax 0.003 0.019
Number of recurrences after chloroquine and primaquine treatment 2 1
DALY weight for severe anemia (due to severe P. vivax or severe hemolysis) 0.112 0.228
Proportion of P. vivax cases that are severe 0.013 0.027
RDT sensitivity 0.90 1.00
DALY weight for P. vivax 0.033 0.081
-0.05 0.00 0.05 0.10 0.15 0.20
Low value High value
D) Base case DALYs averted = 0.024
P. vivax mortality 0 0.001
Number of recurrences after chlorquine treatment 1 8
Length of disability for moderate anemia (due to clinical P. vivax) 0.038 0.086
DALY weight for moderate anemia (due to clinical P. vivax) 0.042 0.167
Relative risk of having at least 1 recurrence if given primaquine 1 0.16
Life expectancy 42 62
Proportion who are pregnant 0.17 0.06
Length of disability for severe anemia 0.083 0.500
Proportion of moderate G6PD abnormal needing blood transfusion 0.050 0.001
Length of disability for clinical P. vivax 0.003 0.019
Number of recurrences after chloroquine and primaquine treatment 2 1
DALY weight for severe anemia (due to severe P. vivax or severe haemolysis) 0.112 0.228
Proportion of P. vivax cases that are severe 0.013 0.027
DALY weight for P. vivax 0.033 0.081
Mortality due to not receiving a transfusion 0.25 0.01
RDT sensitivity for severe G6PD abnormal 0.90 1.00
Proportion not receiving a needed blood transfusion 0.15 0.01
-0.05 0.00 0.05 0.10 0.15
Low value High value
E) Base case cost savings = $7.1
Cost of G6PD RDT $0.88 $10.00
Proportion severe G6PD abnormal 0.21 0.07
Number of recurrences after chlorquine treatment 8 1
Proportion needing blood transfusion for hemolysis 0.150 0.007
Relative risk of having at least 1 recurrence if given primaquine 0.16 1
Cost of hemolytic episode requiring transfusion $402 $166
RDT specificity 0.75 1
Cost of P. vivax visit $11.79 $3.93
RDT sensitivity 1.00 0.90
Cost of observed therapy $2.50 $0.83
Cost of severe P. vivax recurrence $271 $135
Proportion of P. vivax cases that are severe 0.027 0.013
Number of recurrences after chloroquine and primaquine treatment 1 2
Proportion not receiving a needed blood transfusion 0.01 0.15
-$40 -$30 -$20 -$10 $0 $10 $20
Low value High value
F) Base case cost savings = $2.2
Cost of G6PD RDT $0.88 $10.00
Proportion severe G6PD abnormal 0.07 0.02
RDT specificity 0.68 0.99
Number of recurrences after chlorquine treatment 8 1
Proportion of severe G6PD abnormal needing blood transfusion 0.150 0.007
Cost of observed therapy $2.5 $0.8
Relative risk of having at least 1 recurrence if given primaquine 0.16 1
Cost of hemolytic episode requiring transfusion $402 $166
RDT sensitivity for intermediate G6PD abnormal 0.66 0.22
Cost of P. vivax recurrence $11.79 $3.93
Proportion of moderate G6PD abnormal needing blood transfusion 0.050 0.001
Proportion moderate G6PD abnormal 0.24 0.08
RDT sensitivity for severe G6PD abnormal 1.00 0.90
Cost of severe P. vivax recurrence $271 $135
Number of recurrences after chloroquine and primaquine treatment 1 2
Proportion of P. vivax cases that are severe 0.027 0.013
Proportion who are pregnant 0.06 0.17
Proportion not receiving a needed blood transfusion 0.01 0.15
-$40 -$30 -$20 -$10 $0 $10 $20
Low value High value
G) Base case DALYs averted = 0.011
P. vivax mortality 0 0.001
Proportion severe G6PD abnormal 0.07 0.21
Proportion needing blood transfusion for hemolysis 0.007 0.150
Proportion not receiving a needed blood transfusion 0.01 0.15
Number of recurrences after chlorquine treatment 1 8
Life expectancy 39 59
Length of disability for moderate anemia (due to clinical P. vivax) 0.042 0.167
Length of disability for severe anemia 0.083 0.500
DALY weight for moderate anemia (due to clinical P. vivax) 0.038 0.086
RDT sensitivity 0.90 1.00
Relative risk of having at least 1 recurrence if given primaquine 1.00 0.16
DALY weight for severe anemia (due to severe P. vivax or severe hemolysis) 0.112 0.228
Length of disability for clinical P. vivax 0.003 0.019
Number of recurrences after chloroquine and primaquine treatment 2 1
-0.05 0.00 0.05 0.10 0.15
Low value High value
H) Base case DALYs averted = 0.004
Mortality due to not receiving a transfusion 0.01 0.25
P. vivax mortality 0 0.001
Proportion severe G6PD abnormal 0.02 0.07
Proportion not receiving a needed blood transfusion 0.01 0.15
Proportion of severe G6PD abnormal needing blood transfusion 0.007 0.150
Number of recurrences after chlorquine treatment 1 8
Proportion of moderate G6PD abnormal needing blood transfusion 0.001 0.050
Life expectancy 42 62
Length of disability for moderate anemia (due to clinical P. vivax) 0.038 0.086
Proportion who are pregnant 0.17 0.06
Length of disability for severe anemia 0.083 0.500
DALY weight for moderate anemia (due to clinical P. vivax) 0.042 0.167
RDT sensitivity for severe G6PD abnormal 0.90 1.00
Relative risk of having at least 1 recurrence if given primaquine 1 0.16
-0.05 0.00 0.05 0.10 0.15
Low value High value
